# Neoadjuvant pembrolizumab plus nimotuzumab as a bridge to surgery in a frail elderly patient with maxillary gingival and palatal squamous cell carcinoma: a case report

**DOI:** 10.3389/fonc.2026.1993693

**Published:** 2026-10-19

**Authors:** Quanquan Lin, Liang Shang, Zheng-Ze Guo, Feng Wang, Bing Li, Boning Cai, Lin Feng, Haizhong Zhang, Zhigang Song, Zhiyong Wu, Qing Xi

**Affiliations:** 1 Department of Stomatology, The First Medical Center of People's Liberation Army (PLA) General Hospital, Beijing, China; 2 Department of Stomatology, The Sixth Medical Center of People's Liberation Army (PLA) General Hospital, Beijing, China; 3 Department of Ambulatory Medicine, The First Medical Center of People's Liberation Army (PLA) General Hospital, Beijing, China; 4 Department of Stomatology, The 81st Group Army Hospital of the Chinese People’s Liberation Army, Zhangjiakou, Hebei, China; 5 Department of Radiation Oncology, The First Medical Center of People's Liberation Army (PLA) General Hospital, Beijing, China; 6 Department of Pathology, The First Medical Center of People's Liberation Army (PLA) General Hospital, Beijing, China

**Keywords:** case report, frailty, head and neck squamous cell carcinoma, malnutrition, neoadjuvant therapy, nimotuzumab, oral squamous cell carcinoma, pathological complete response

## Abstract

**Background:**

In older patients with oral squamous cell carcinoma, tumor-related symptoms can impair oral intake and compromise fitness for surgery. This case describes an individualized chemotherapy-free bridge-to-surgery approach that combined tumor-directed treatment with nutritional and functional support.

**Case presentation:**

A 75-year-old woman presented with left maxillary gingival and hard-palatal squamous cell carcinoma, severe oral pain, dysphagia, a 5-kg weight loss over 2 weeks, and an Eastern Cooperative Oncology Group performance status of 3. Biopsy confirmed well-differentiated squamous cell carcinoma with positive epidermal growth factor receptor expression and a programmed death ligand 1 combined positive score of 70. Because cisplatin was considered unsuitable at presentation, she received pembrolizumab (200 mg every 3 weeks) plus nimotuzumab (200 mg weekly), with concurrent supportive care. After two treatment cycles, oral intake recovered, body weight increased from 70 to 75 kg, and performance status improved from 3 to 1. Computed tomography showed marked regression, reported as a partial response. Maxillectomy and left neck dissection were performed on 12 December 2025. Postoperative pathology was reported as a complete response (ypT0N0), with 0 of 32 lymph nodes involved. Postoperative maintenance immunotherapy remained ongoing, and no recurrence was reported at the last follow-up in September 2026.

**Conclusion:**

This case illustrates the feasibility of preoperative pembrolizumab plus nimotuzumab with concurrent supportive care as a bridge to definitive surgery in one clinically frail older patient. It does not establish the efficacy or comparative safety of pembrolizumab plus nimotuzumab, the independent contribution of either drug, or the durability of disease control. The bridge-to-surgery concept warrants prospective evaluation in carefully selected patients.

## 1 Introduction

Based on GLOBOCAN 2024, the combined estimates for cancers of the lip and oral cavity, oropharynx, nasopharynx, hypopharynx, and larynx were approximately 965,000 new cases and 454,000 deaths worldwide (1). Lip and oral cavity cancers accounted for 452,205 new cases and 194,108 deaths, underscoring their substantial contribution to this burden (1).

Oral squamous cell carcinoma (OSCC) can compromise nutrition through pain, ulceration, bleeding, and impaired oral intake. In older patients, these problems may coexist with substantial functional vulnerability (2). Perioperative malnutrition has been associated with adverse outcomes after head and neck cancer surgery (3). Surgical resection remains central to curative treatment for resectable oral cavity cancer, with postoperative radiotherapy or cisplatin-based chemoradiotherapy selected according to pathological risk (4). In the adjuvant setting, concurrent cisplatin may be unsuitable for patients unable to tolerate platinum-based therapy or with cisplatin-specific contraindications, including clinically significant renal impairment, hearing loss, peripheral neuropathy, or poor performance status (4, 5).

For a patient whose tumor is anatomically resectable but whose physiological reserve is inadequate, the immediate objective may be to restore fitness for definitive treatment. In this report, “bridge to surgery” refers to tumor neoadjuvant therapy treatment delivered alongside supportive care, followed by reassessment of symptoms, oral intake, performance status, and surgical candidacy. It does not imply that systemic therapy replaces resection or that tumor regression alone justifies less extensive surgery.

Neoadjuvant and perioperative programmed cell death protein 1 (PD-1) blockade have demonstrated clinical activity in resectable head and neck squamous cell carcinoma (HNSCC), including improved event-free survival with perioperative pembrolizumab added to standard care in KEYNOTE-689 (6, 7). The trial included older adults, with ages extending to 82 and 87 years in the pembrolizumab and control groups, respectively, but required Eastern Cooperative Oncology Group (ECOG) performance status 0–1 and eligibility for primary surgery (7). Surgery was completed in approximately 88% of participants in each group (7). These overall findings support feasibility in a selected population but should not be interpreted as elderly-specific tolerability data or extrapolated directly to clinically frail patients presenting with ECOG performance status 3.

Previous studies have evaluated pembrolizumab plus the epidermal growth factor receptor (EGFR) antibody cetuximab in recurrent or metastatic HNSCC, and nimotuzumab with cisplatin-based chemoradiotherapy in locally advanced disease (8, 9). More directly, an 86-patient real-world study by Li et al. reported favorable response and survival outcomes with pembrolizumab plus nimotuzumab compared with pembrolizumab alone in recurrent, unresectable, or metastatic HNSCC (10). These findings support further investigation of this combination but do not establish its efficacy or perioperative safety in frail surgical candidates. We describe a clinically frail older woman whose nutritional and functional status improved during this chemotherapy-free combination and concurrent supportive care, allowing subsequent maxillectomy and neck dissection.

## 2 Case description

### 2.1 Patient information and clinical findings

A 75-year-old Han Chinese woman presented with a progressively enlarging lesion of the left maxillary gingiva extending to the hard palate and buccal region (**Figure 1**). Intermittent bleeding, ulceration, severe chewing-related pain, and dysphagia had markedly reduced her oral intake. She also reported a painless enlarging left neck mass. Before referral, the lesion had been treated as an oral ulcer with oral anti-inflammatory medication, without improvement. She had never smoked and reported no alcohol use. Her medical history included atrial fibrillation and hypertension, reported for 12 years. Amlodipine and benazepril was listed among her medications. No relevant hereditary history or known drug allergy was reported.

**Figure 1:**
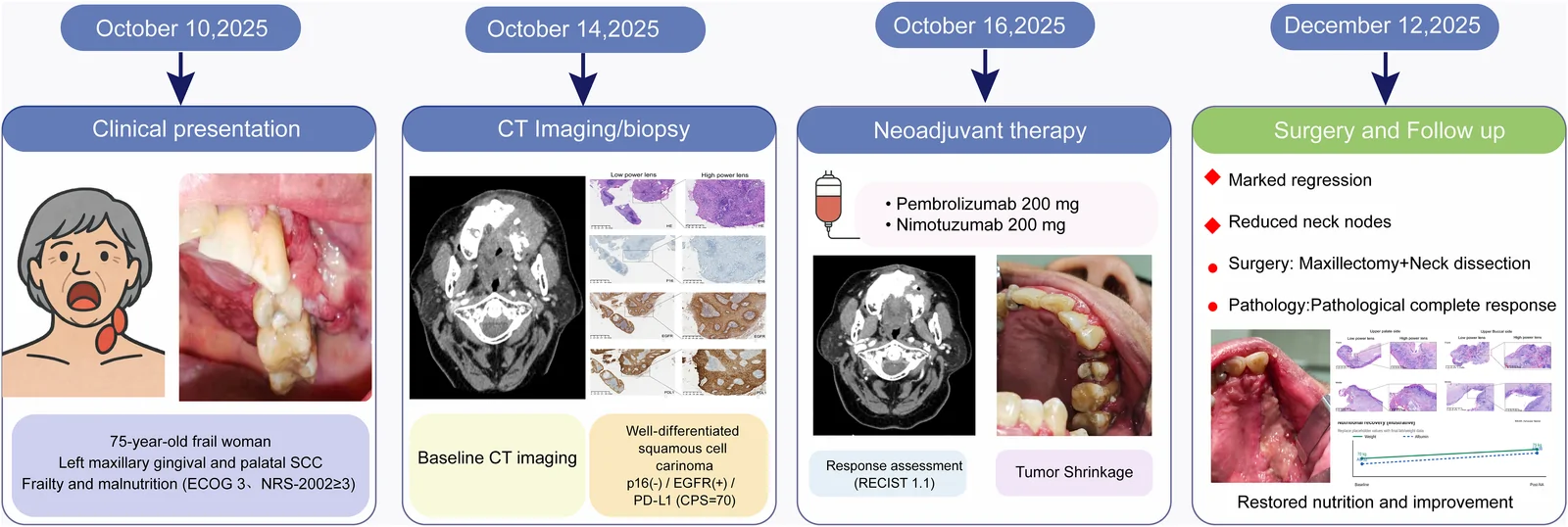
Clinical and radiological course before definitive surgery. Pretreatment and post-neoadjuvant clinical and CT findings in a 75-year-old woman with left maxillary gingival and hard-palatal squamous cell carcinoma. The baseline lesion caused severe oral pain, impaired oral intake, and functional decline. After the reported two-cycle course of pembrolizumab plus nimotuzumab with supportive care, the intraoral lesion and cervical lymphadenopathy regressed, oral intake improved, and ECOG performance status improved from 3 to 1. CT response was reported as partial by RECIST 1.1. The clinical timeline is provided in **Table 1**.

At presentation, Eastern Cooperative Oncology Group (ECOG) performance status was recorded as 3 in the setting of severe oral pain, dysphagia, markedly reduced oral intake, and a decrease in body weight from 75 to 70 kg over approximately 2 weeks, despite a body mass index of 26.7 kg/m². The functional assessment by the treating clinical team at initial presentation documented limited self-care, a need for assistance with daily activities, and confinement to a bed or chair for more than half of waking hours. Nutritional Risk Screening 2002 (NRS-2002) indicated high nutritional risk (11).

Frailty was assessed clinically by the treating clinical team, based on a clinical review of the patient’s nutritional status, functional capacity, and cardiovascular comorbidities. The clinical context included rapid weight loss, reduced oral intake, functional limitation, and cardiovascular comorbidities. No baseline score from a validated frailty instrument was documented. In this report, “clinically assessed frailty” therefore denotes an unstandardized clinical impression of vulnerability and reduced physiological reserve rather than a formally graded frailty syndrome. Intraoral examination showed an ulcerated, erythematous lesion extending from the left maxillary canine/premolar region to the tuberosity, with adjacent tooth mobility. Mouth opening was approximately three finger breadths, without obvious trismus. A mobile, non-tender left neck cervical node measured approximately 3 * 2 * 3 cm.

Baseline serum albumin was 32 g/L, prealbumin 125 mg/L, hemoglobin 110 g/L, lymphocyte count 0.90 × 10^9^/L, and C-reactive protein (CRP) 38 mg/L. These laboratory findings accompanied the clinical decline. Serial laboratory values are presented in **Supplementary Table S1**. The clinical timeline is summarized in **Table 1**.

**Table 1:** Clinical timeline and key treatment-related observations.

Time point	Clinical event or observation
10 October 2025	Presentation with progressive left maxillary gingival/hard-palatal disease, severe pain and reduced oral intake; approximately 5-kg weight loss over 2 weeks; ECOG 3 and high nutritional risk. Albumin 32 g/L, prealbumin 125 mg/L, CRP 38 mg/L.
14 October 2025	Baseline imaging and biopsy work-up. Approximately 44 × 42-mm maxillary lesion with bone destruction and suspicious ipsilateral cervical nodes. Biopsy showed well-differentiated SCC; p16 negative, EGFR positive, PD-L1 CPS 70.
16 October 2025	Pembrolizumab 200 mg every 3 weeks plus nimotuzumab 200 mg weekly initiated, with concurrent nutritional, symptom-directed and functional support.
After 1 cycle	Albumin 35 g/L, prealbumin 180 mg/L and CRP 16 mg/L. Other serial laboratory values are provided in **Supplementary Table S1**.
After 2 cycles; November 2025	Improved oral symptoms and independent oral intake; body weight 75 kg and ECOG 1. Albumin 38 g/L, prealbumin 235 mg/L and CRP 6 mg/L.
December 2025, Before surgery	CT restaging showed marked primary-tumor regression and reduced cervical lymphadenopathy; partial response was reported under RECIST 1.1.
12 December 2025	Maxillectomy and left neck dissection of levels Ia–IV. Frozen-section margins were negative. No flap reconstruction was performed.
December 2025, Postoperative	Pathology was reported as ypT0N0, with 0/32 nodes involved. The description of degenerative epithelial clusters requires confirmation of cell viability against the original pathology record.
1 month after surgery	Follow-up assessment was performed; maintenance immunotherapy continued.
3 months after surgery	Follow-up assessment was performed; maintenance immunotherapy continued.
6 months after surgery	Follow-up assessment was performed; maintenance immunotherapy continued.
September 2026	Maintenance immunotherapy remained ongoing at the last follow-up in September 2026. No recurrence was reported.

CPS, combined positive score; CRP, C-reactive protein; CT, computed tomography; ECOG, Eastern cooperative oncology group; EGFR, epidermal growth factor receptor; PD-L1, programmed death ligand 1; RECIST, Response evaluation criteria in solid tumors; SCC, squamous cell carcinoma.

### 2.2 Diagnostic assessment

Incisional biopsy confirmed well-differentiated squamous cell carcinoma. Immunohistochemistry showed negative p16, positive EGFR, and a programmed death ligand 1 (PD-L1) combined positive score (CPS) of 70 (**Figure 2**). Baseline contrast-enhanced computed tomography (CT) showed an approximately 44*42mm left maxillary lesion with heterogeneous enhancement, extension into adjacent facial soft tissue, and destruction of the anterior and inferior maxillary sinus walls. Multiple ipsilateral cervical nodes were enlarged and considered suspicious. The pretreatment clinical stage was recorded as cT4aN1M0 under the eighth edition of the American Joint Committee on Cancer staging system. At the pretreatment assessment, the tumor was considered anatomically resectable. Immediate surgery was deferred because of concerns about the patient’s ability to tolerate extensive resection and flap reconstruction, rather than an anatomical barrier to complete tumor removal.

**Figure 2:**
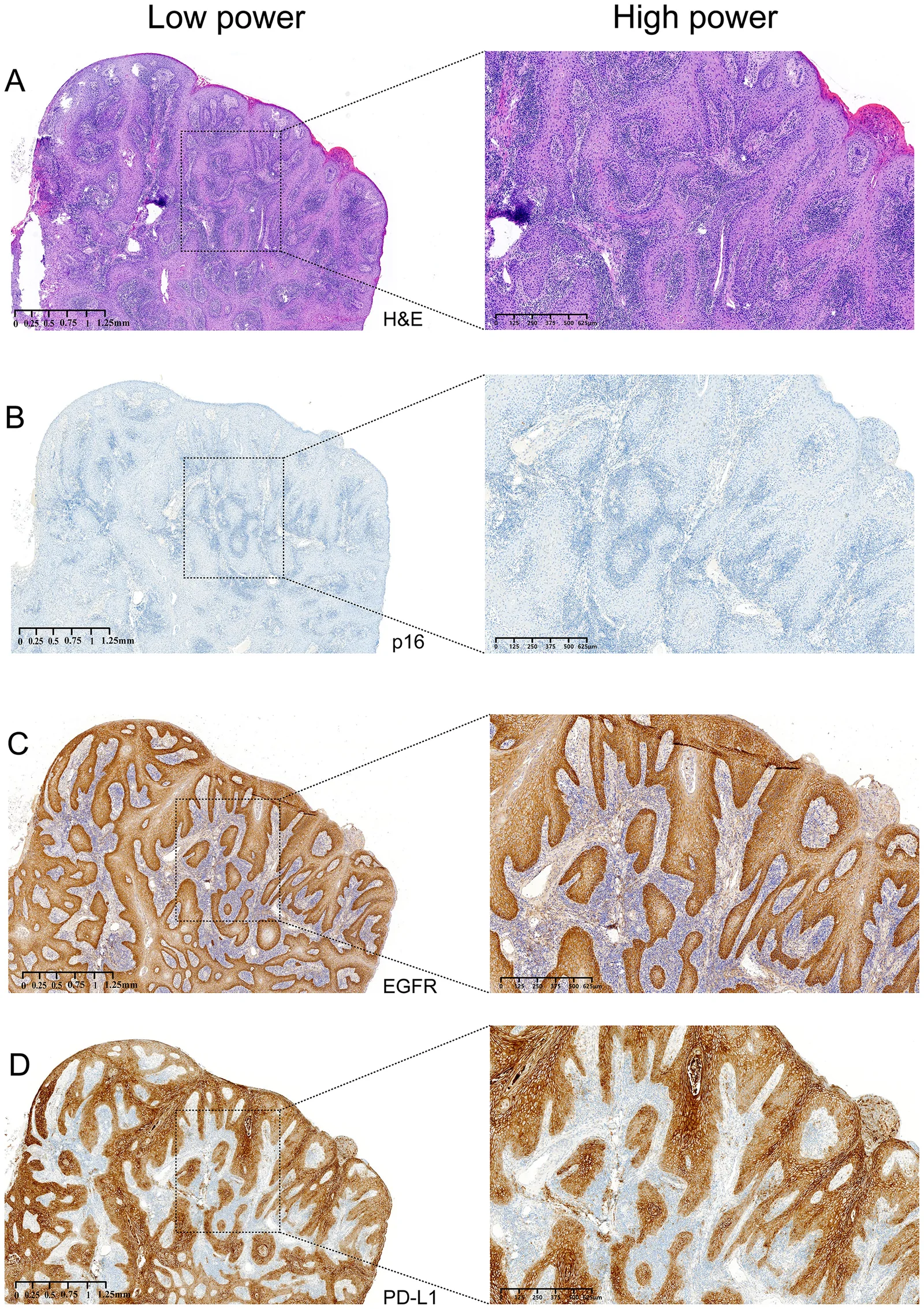
Pretreatment histopathological and immunohistochemical findings. **(A)** Hematoxylin and eosin (H&E)-stained biopsy sections showing well-differentiated squamous cell carcinoma. Immunohistochemistry was reported as **(B)** p16 negative, **(C)** EGFR positive, and **(D)** PD-L1 CPS 70. Panel labels, staining details, and scale bars require confirmation against the original images.

The multidisciplinary team considered cisplatin unsuitable at presentation because of poor performance status, nutritional compromise, clinically assessed frailty, and cardiovascular comorbidities. The main treatment-planning difficulty was the perceived risk of immediate extensive surgery in a patient with limited physiological reserve. The aim was therefore to control tumor-related symptoms while improving fitness for curative-intent resection.

At presentation, the patient’s advanced age, difficulty with oral intake, rapid tumor progression, and progressively deteriorating general condition raised concerns about her ability to tolerate extensive surgery with flap reconstruction. Despite ECOG performance status 3, the team therefore selected an individualized preoperative immunotherapy-based approach, together with supportive care, to control tumor progression and potentially improve fitness for surgery. The plan was to reassess tumor size and overall clinical status serially and to proceed promptly to definitive surgery as soon as clinically feasible. The intended goal was to enable a less extensive and less morbid operation, including a reduced reconstructive burden, while retaining complete tumor resection as the oncological objective.

### 2.3 Therapeutic intervention

Treatment began on 16 October 2025. The reported regimen was pembrolizumab 200 mg every 3 weeks plus nimotuzumab 200 mg weekly, without cytotoxic chemotherapy. Two treatment cycles were reported. The choice of PD-1/EGFR targeting was individualized by the multidisciplinary team in the context of high PD-L1 expression, EGFR positivity, and limited tolerance for cytotoxic therapy. No grade ≥2 immune-related adverse events or infusion reactions were reported during the neoadjuvant period.

Supportive care was provided concurrently and included structured nutritional support, analgesia and oral care to facilitate intake, monitoring and correction of fluid or electrolyte disturbances, cardiovascular optimization, and functional prehabilitation as tolerated. Clinical symptoms, body weight, laboratory results, and performance status were reassessed during treatment. Radiological response was reported using Response Evaluation Criteria in Solid Tumors (RECIST), version 1.1 (12).

### 2.4 Treatment response and definitive surgery

After two cycles, oral pain had largely resolved and the patient could eat independently. Body weight increased from 70 to 75 kg, and ECOG performance status improved from 3 to 1. Albumin increased to 38 g/L and prealbumin to 235 mg/L, while CRP decreased to 6 mg/L. No gross tumor was visible in the previously involved intraoral area. CT restaging showed marked regression of the primary lesion and reduction in cervical lymphadenopathy, reported as a partial response by RECIST 1.1 (**Figure 1**).

Following improvement in nutritional and functional status, the patient underwent maxillectomy and left neck dissection of levels Ia, Ib, II, III, and IV on 12 December 2025. Intraoperative frozen-section margins were negative. No flap reconstruction was performed; the recorded considerations were advanced age and limited physiological reserve.

### 2.5 Pathological assessment and follow-up

Final pathology was reported as a pathological complete response (pCR; ypT0N0). Serial sections sampled the anterior, middle, and posterior regions of the former tumor bed on its palatal and buccal aspects. The postoperative narrative described no residual viable squamous cell carcinoma in the examined tumor bed or maxillary bone, with dense fibrosis, granulation tissue, chronic inflammatory infiltration, histiocytes/foamy macrophages, and occasional multinucleated giant cells (**Figure 3**). The accompanying pathological summary also described scattered degenerative atypical epithelial cell clusters interpreted as regressed carcinoma.

**Figure 3:**
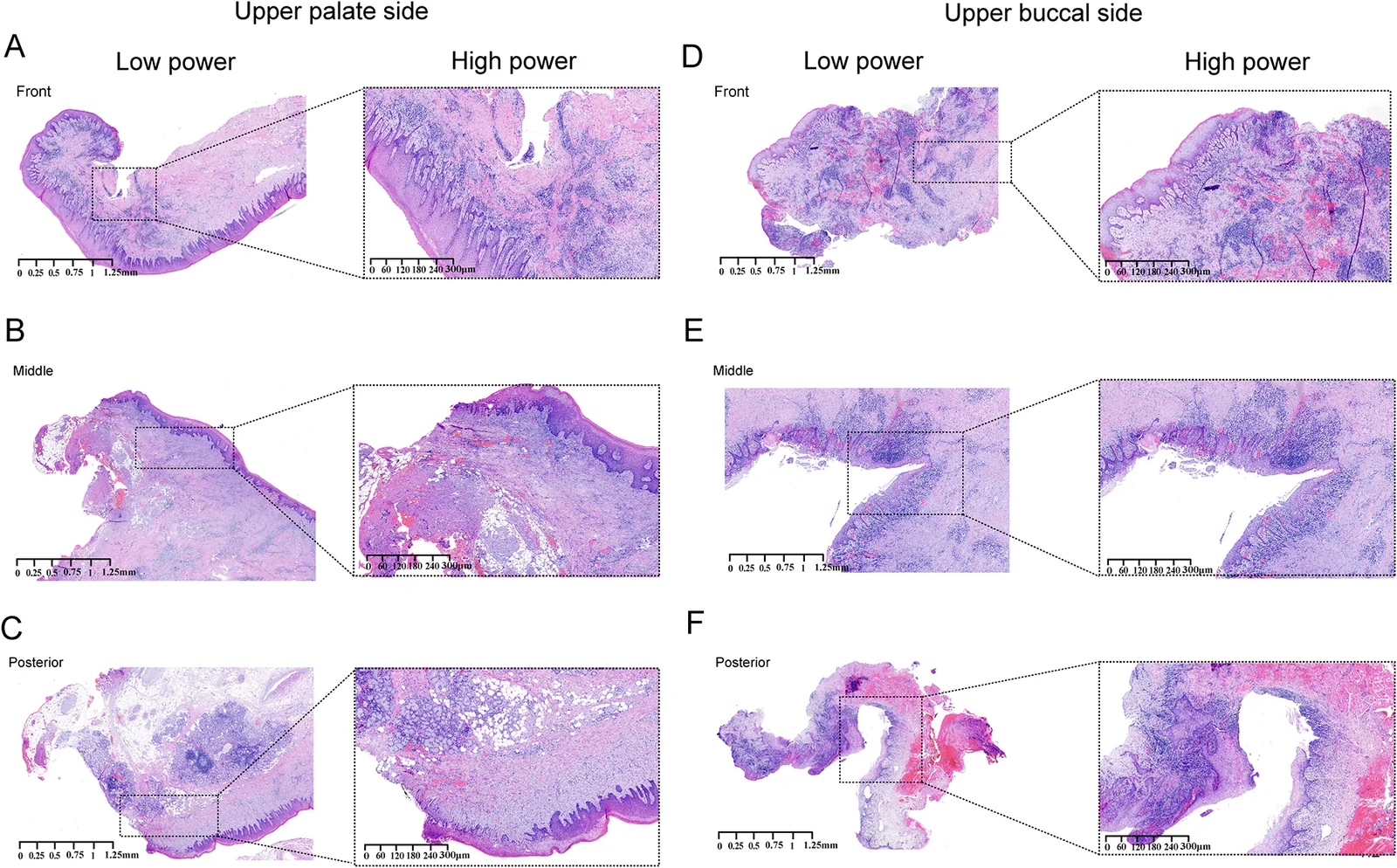
Post-treatment histopathological findings in the resected tumor bed. H&E-stained sections from the palatal **(A–C)** and buccal **(D–F)** aspects of the former tumor bed, sampled at anterior **(A, D)**, middle **(B, E)**, and posterior **(C, F)** locations. The postoperative narrative described fibrosis, granulation tissue, chronic inflammation, histiocytes/foamy macrophages, and occasional multinucleated giant cells, without residual viable squamous cell carcinoma. The original pathological summary also described degenerative atypical epithelial clusters; their viability and the final response classification must be confirmed before the figure is finalized. The reported nodal findings (0/32) are described separately in the case text.

All 32 dissected cervical lymph nodes were negative for metastatic carcinoma: 0/10 at levels Ia–Ib, 0/10 at level II, 0/4 at level III, and 0/8 at level IV. One node showed necrosis and dystrophic calcification, described as compatible with treatment effect. Pretreatment nodal involvement was suspected radiologically rather than confirmed by a nodal biopsy in the available case description.

Following surgery, the case was reviewed by the multidisciplinary team. Given the negative resection margins and the absence of metastatic carcinoma in all 32 dissected cervical lymph nodes, postoperative radiotherapy and cytotoxic chemotherapy were not recommended. The patient received postoperative maintenance immunotherapy, which remained ongoing at the last follow-up in September 2026. Close surveillance was maintained, with follow-up assessments performed at 1, 3, and 6 months after surgery. No recurrence was reported at the last follow-up, approximately 9 months after surgery.

## 3 Discussion

The main educational value of this case is the recovery of access to curative-intent surgery, rather than pCR alone. At presentation, the patient had severe oral symptoms, rapid weight loss, and ECOG performance status 3. During tumor-directed treatment and supportive care, oral intake, weight, and performance status improved sufficiently for the planned resection to be performed. This temporal sequence is consistent with a bridge-to-surgery concept, but it cannot show that the drug combination was necessary or that surgery would otherwise have remained impossible.

The improvement in oral symptoms, intake, and ECOG performance status during treatment suggests that the initial functional decline included a potentially reversible component. This is an interpretation of the observed clinical course, not evidence that pain alone explained the baseline ECOG score or that physiological reserve was otherwise adequate. Because tumor-directed treatment and supportive care were provided concurrently, their contributions to functional recovery cannot be separated. This individual course therefore does not establish the safety of the regimen in an unselected population with ECOG performance status 3.

The patient’s course also illustrates why host recovery should be assessed alongside tumor response. Cancer nutrition guidance supports repeated evaluation of intake, weight change, and nutritional risk, with nutritional care integrated into oncological treatment (2). Here, improved oral intake and ECOG status were clinically relevant observations. Rising albumin and prealbumin should not, however, be treated as independent proof that malnutrition or muscle depletion resolved: these proteins are influenced by inflammation and are not stand-alone measures of nutritional status (13). The parallel fall in CRP therefore requires a combined interpretation of the biochemical changes. No conclusion about recovery of muscle mass can be drawn from the available measurements.

Perioperative immunotherapy provides a relevant context but not direct validation of the present regimen. In the phase II study by Wise-Draper et al., neoadjuvant pembrolizumab was followed by surgery and risk-adapted adjuvant treatment (6). KEYNOTE-689 subsequently demonstrated improved event-free survival when neoadjuvant and adjuvant pembrolizumab were added to surgery and postoperative radiotherapy, with or without cisplatin (7). Those results concern a complete perioperative strategy, not two preoperative cycles of pembrolizumab plus nimotuzumab followed by surgery and postoperative maintenance immunotherapy without radiotherapy (7). A matched cohort reported by Tang et al. found no increase in most evaluated postoperative adverse events after neoadjuvant pembrolizumab in oral cavity cancer, but this does not establish the safety of a different combination in a patient presenting with ECOG performance status 3 (14).

The rationale for dual PD-1/EGFR targeting requires similar caution. Sacco et al. reported antitumor activity with pembrolizumab plus cetuximab in recurrent or metastatic HNSCC, providing adjacent clinical evidence rather than proof for nimotuzumab in a neoadjuvant setting (8). Patil et al. evaluated nimotuzumab added to cisplatin-based chemoradiotherapy, not a chemotherapy-free surgical bridge (9). These distinctions preclude treating the antibodies or treatment settings as interchangeable. In this patient, CPS 70 and EGFR positivity informed the treatment rationale, but neither the observed response nor this biomarker combination establishes a validated selection rule. Because both drugs and supportive care were introduced together, their individual contributions cannot be determined.

A previous OSCC case reported by Olmos et al. described substantial histological regression after a single preoperative pembrolizumab dose before salvage surgery, with viable tumor still present (15). That report concerned recurrent disease in a much younger patient and differs from the present focus on recovering surgical fitness in an older patient with nutritional and functional compromise (14). Both cases place surgery and tissue assessment within the treatment pathway; neither establishes that clinical regression is sufficient to omit definitive local treatment.

Pathological interpretation is especially important in a report centered on a complete response. In this case, a radiological partial response coexisted with a postoperative narrative reporting no viable tumor, illustrating that radiological and pathological endpoints describe different observations. The simultaneous description of degenerative atypical epithelial clusters must be reconciled with the original slides and final pathology report before pCR is regarded as unequivocally established. Furthermore, 0/32 nodes after treatment does not by itself prove clearance of histologically confirmed nodal metastases, because baseline nodal disease was not biopsy-confirmed in the supplied description. The case provides no evidence for reducing resection margins, omitting neck dissection, or withholding indicated postoperative treatment on the basis of response.

Although negative resection margins and node-negative pathology informed the postoperative MDT decision, this single case does not validate response-based omission of postoperative radiotherapy. Similarly, the administration of postoperative maintenance immunotherapy should not be interpreted as evidence that it can replace otherwise indicated radiotherapy.

Translational studies suggest potentially complementary effects of pembrolizumab and nimotuzumab on the tumor immune microenvironment. In HNSCC treated with neoadjuvant pembrolizumab, Oliveira et al. linked pathological response to reactivation of pre-existing tumor-resident CD8^+^ T cells with cytotoxic potential (16). In preclinical HNSCC models, Hu et al. found that nimotuzumab inhibited TGF-β-induced CD276 expression through EGFR/MEK/ERK blockade, accompanied by increased intratumoral cytotoxic and helper T-cell frequencies (17). Complementary *in vitro* work by Mazorra et al. demonstrated nimotuzumab-mediated antibody-dependent cellular cytotoxicity and natural killer–dendritic cell interactions that promoted EGFR-specific CD8^+^ T-cell priming (18). Together, these findings support the hypothesis that the combination may enhance antitumor T-cell activity while modifying immune-suppressive signaling and antigen-specific immune priming; they do not, however, establish synergy between the two agents (16–18).

Several limitations restrict interpretation. This is a single, selected case without a comparator. Frailty was assessed clinically without a prospectively recorded validated instrument. This limits reproducibility and the distinction between pre-existing frailty and acute disease-related functional decline. The subsequent improvement in ECOG performance status should not be equated with documented reversal of frailty.

In the present case, the resected tumor bed showed fibrosis and inflammatory infiltration. However, this report does not include paired quantitative immune profiling. These histological observations therefore cannot establish changes in specific immune-cell populations, confirm the proposed signaling mechanisms, or determine the contribution of either drug to the response.

The exact NRS-2002 score, detailed nutritional prescription, complete drug-administration record, and quantitative post-treatment imaging measurements were not provided. Detailed postoperative morbidity and objective functional outcomes are not fully described. Follow-up is short, and no inference regarding survival benefit or durable disease control is possible. These gaps are particularly relevant when the proposed clinical benefit is improved treatment feasibility rather than tumor shrinkage alone.

Future evaluation of this approach should assess both tumor control and restoration of treatment fitness. Relevant observations would include treatment completion, nutritional intake, physical function, time to surgery, operative morbidity, pathological response, postoperative treatment delivery, and longer-term disease control. Such evaluation would need to distinguish a supportive bridge for physiologically vulnerable patients from an indication to intensify systemic therapy or de-escalate standard local treatment.

## 4 Conclusion

This case illustrates the feasibility of preoperative pembrolizumab plus nimotuzumab with concurrent supportive care as a bridge to definitive surgery in one clinically frail older patient. It does not establish the efficacy or comparative safety of the combination, the independent contribution of either drug, or the durability of disease control. The bridge-to-surgery concept warrants prospective evaluation in carefully selected patients.

## 5 Patient perspective (Family-reported account)

The following account was provided by the patient’s son and reflects the family’s observations rather than a directly obtained patient-reported narrative. During preparation of the original report, the narrative was collected from her son, and a separate interview specifically seeking the patient’s own perspective was not conducted. Her son described severe oral pain and difficulty eating before treatment, accompanied by weight loss, weakness, and declining confidence about surgery. During neoadjuvant treatment and supportive care, the patient resumed oral intake and gradually regained strength. The family felt reassured when surgery became feasible and reported relief after discussion of the postoperative pathological findings. The patient and family agreed to continued surveillance.

## Data Availability

The original contributions presented in the study are included in the article/**Supplementary Material**. Further inquiries can be directed to the corresponding authors.
