# Supplementary material for: Neoadjuvant pembrolizumab plus nimotuzumab as a bridge to surgery in a frail elderly patient with maxillary gingival and palatal squamous cell carcinoma: a case report
Source: Front Oncol. 2026 Oct 19;16:1993693. doi: 10.3389/fonc.2026.1993693 (PMC13724471; doi:10.3389/fonc.2026.1993693)
Supplement: Supplementary file 1 [file Table1.docx]

# Supplementary Table S1. Serial laboratory values during neoadjuvant treatment and supportive care.

| **Laboratory variable** | **Baseline** | **After 1 cycle** | **After 2 cycles** | **Reference range** |
| --- | --- | --- | --- | --- |
| Albumin (g/L) | 32 | 35 | 38 | 35–55 |
| Prealbumin (mg/L) | 125 | 180 | 235 | 200–400 |
| Hemoglobin (g/L) | 110 | 137 | 144 | 115–150 (female) |
| Lymphocyte count (×10⁹/L) | 0.90 | 1.48 | 1.32 | 1.1–3.2 |
| C-reactive protein (mg/L) | 38 | 16 | 6 | <8 |
| Sodium (mmol/L) | 134 | 137 | 139 | 135–145 |
| Potassium (mmol/L) | 3.5 | 3.85 | 3.9 | 3.5–5.3 |

Values and reference ranges are transcribed from the source manuscript. Cycle-based time points are retained because exact blood-sampling dates were not provided. No missing values or post-treatment dates have been imputed.

Albumin and prealbumin should be interpreted with clinical intake, body-weight change, and inflammatory status rather than as stand-alone measures of nutritional recovery; see reference 11 in the main manuscript.
